# Cold exposure induces dynamic, heterogeneous alterations in human brown adipose tissue lipid content

**DOI:** 10.1038/s41598-019-49936-x

**Published:** 2019-09-19

**Authors:** Crystal L. Coolbaugh, Bruce M. Damon, Emily C. Bush, E. Brian Welch, Theodore F. Towse

**Affiliations:** 1 Vanderbilt University Institute of Imaging Science, Vanderbilt University Medical Center, Nashville, TN USA; 2 Department of Radiology and Radiological Sciences, Vanderbilt University Medical Center, Nashville, TN USA; 3 Department of Biomedical Engineering, Vanderbilt University, Nashville, TN USA; 4 Department of Molecular Physiology and Biophysics, Vanderbilt University, Nashville, TN USA; 5 Department of Physical Medicine and Rehabilitation, Vanderbilt University Medical Center, Nashville, TN USA; 6 Department of Biomedical Sciences, Grand Valley State, Allendale, MI USA

**Keywords:** Magnetic resonance imaging, Fat metabolism

## Abstract

Brown adipose tissue undergoes a dynamic, heterogeneous response to cold exposure that can include the simultaneous synthesis, uptake, and oxidation of fatty acids. The purpose of this work was to quantify these changes in brown adipose tissue lipid content (fat-signal fraction (FSF)) using fat-water magnetic resonance imaging during individualized cooling to 3 **°**C above a participant’s shiver threshold. Eight healthy men completed familiarization, perception-based cooling, and MRI-cooling visits. FSF maps of the supraclavicular region were acquired in thermoneutrality and during cooling (59.5 ± 6.5 min). Brown adipose tissue regions of interest were defined, and voxels were grouped into FSF decades (0–10%, 10–20%…90–100%) according to their initial value. Brown adipose tissue contained a heterogeneous morphology of lipid content. Voxels with initial FSF values of 60–100% (*P* < 0.05) exhibited a significant decrease in FSF while a simultaneous increase in FSF occurred in voxels with initial FSF values of 0–30% (*P* < 0.05). These data suggest that in healthy young men, cold exposure elicits a dynamic and heterogeneous response in brown adipose tissue, with areas initially rich with lipid undergoing net lipid loss and areas of low initial lipid undergoing a net lipid accumulation.

## Introduction

Human brown adipose tissue exhibits a variety of neural, vascular, and metabolic responses to cold exposure. For example, cold exposure stimulates a sympathetically mediated increase in the rate of oxidative metabolism of brown and beige adipocytes. This “activation” of brown and beige fat results in increased uptake of both glucose and non-esterified, or free, fatty acids. Due to the elevated expression of uncoupling protein-1 in the mitochondria of these adipocytes, the increased rate of oxidative metabolism also generates heat in an effort to defend core body temperature^1,2^. While brown adipose tissue was initially rediscovered in adult humans using ^18^F-deoxyglucose positron emission tomography (FDG-PET)^3–5^, quantitatively the most important substrate for brown adipose tissue thermogenesis is intracellular lipids^6–10^. Indeed, cold-activated brown adipocytes exhibit a complex set of behaviors regarding lipid mobilization, with the synthesis, uptake, and oxidation of fatty acids occurring simultaneously^11,12^.

Biomedical imaging and spectroscopy are preeminent methods for studying the spatial distribution of many physiological and biochemical processes *in vivo*, and they provide complementary information about the structure and function of brown adipose tissue^13,14^. Of these methods, PET, X-ray computed tomography, and magnetic resonance (MR) imaging and spectroscopy allow investigators to study brown adipose tissue lipid content. PET imaging of tracers such as ^18^F-6-thia-heptadecanoic acid can be used to measure the uptake of non-esterified fatty acids into brown adipose tissue during cold exposure^7,15–17^. X-ray computed tomography can distinguish between brown and white adipose tissues based on their Hounsfield units^18,19^. ^1^H-MR spectroscopy has been used to characterize the content and degree of unsaturation of the lipids within brown vs. white adipocytes, revealing reduced levels of unsaturation and polyunsaturation in the lipids stored in brown adipose tissue^20^. Quantitative fat-water MRI, originally described as Dixon imaging^21^, allows the separation of fat- and water-derived MRI signal components and the spatial mapping of fat content in organs such as white and brown adipose tissue, skeletal muscle, and liver^22^. Typically, this is expressed as the fat signal fraction (FSF), which is the proportion of MRI signal that is derived from lipids. Importantly, FSF can be used to detect brown adipose tissue independent of its activation status meaning that cold exposure is not required to estimate brown adipose tissue’s distribution in the body^19^.

Another important advantage of MRI is that it is non-invasive, non-destructive, and absent of ionizing radiation. Although this property allows MRI to be used to study the temporal dynamics of physiological processes *in vivo*, we are aware of only a single study in which MRI was used to study FSF changes as a function of time during cold exposure^23^. However, this study used a two-point Dixon imaging method, which is unable to account for natural signal decay processes, inhomogeneity in the MRI scanner’s static magnetic field, or the existence of multiple lipid moieties in lipid molecules as it estimates FSF^24,25^. The use of MRI to study human brown adipose tissue is also limited by the lack of uniformity in data acquisition and analysis methods. For example, there is a lack of consensus concerning the range of FSF values that define human brown adipose tissue^13^ with the ideal range apparently varying in a subject-specific manner^26^.

Therefore, the overall goal of this study was to measure the response of human brown adipose tissue with fat-water MRI during approximately one hour of personalized cooling to a target temperature of 3 **°**C above a participant’s previously determined shiver threshold^27^. Our primary hypothesis was that brown adipose tissue lipid content, represented as FSF, would decrease following cold exposure. We also sought to characterize the temporal relationship between brown adipose tissue FSF and cold stress. We find that in healthy, young adult males, supraclavicular brown adipose tissue contains a diverse morphological distribution of lipids that undergoes heterogeneous changes in brown adipose tissue FSF in response to a cold stimulus.

## Methods

### Participants

Representative data from Stahl *et al*.^23^ were used to determine the number of participants required to detect a change in brown adipose tissue FSF in response to individualized cooling. Assuming a decrease of 2.9% (effect size = 1.45) to be scientifically relevant, a minimum sample size of 7 volunteers was needed to compare thermoneutral and cold exposure brown adipose tissue FSF values with α = 0.05 and β = 0.85^28^. An additional subject was recruited to account for attrition.

Participants were recruited for the study from the local community via word-of-mouth and email advertisements. Prior to enrollment, volunteers completed a telephone screening interview to ensure compatibility with the following eligibility criteria: between 18 and 35 years of age; no use of tobacco products; no history or symptoms of cardiovascular, pulmonary, neurological, or metabolic disease; no current use of prescribed or over-the-counter medications known to affect thermoregulation or brown adipose tissue activity^29,30^; and no contraindications for an MRI exam. Subject recruitment was not targeted towards a specific physical activity level or racial or ethnic group. Female volunteers were excluded from this study due to difficulties scheduling multiple study visits during the follicular phase of the menstrual cycle, a constraint needed to limit the potential effects of sex hormones on thermoregulation^31^. The Vanderbilt University Medical Center Institutional Review Board approved the study procedures. All participants provided written, informed consent, and methods were carried out in accordance with relevant guidelines and regulations.

### Study procedures

**Figure 1:**
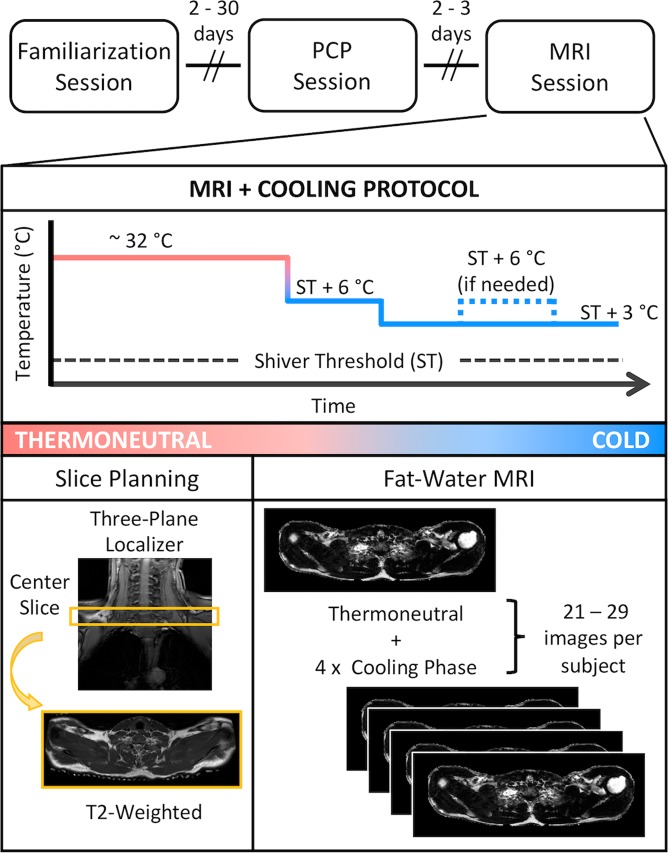
Schematic of the study timeline and procedures. Familiarization and perception-based cooling protocol (PCP) sessions were completed as previously described^27^. The magnetic resonance image (MRI) session included imaging procedures (slice planning and fat-water MRI) during thermoneutral and cold conditions. The temperature of two water-circulating blankets was adjusted to individualize cooling according to the participant’s shiver threshold (ST), which was identified during the PCP session.

Volunteers completed three separate study visits: familiarization, a perception-based cooling protocol (PCP), and an MRI cooling protocol (Fig. 1). Prior to PCP and MRI sessions, participants were required to avoid vigorous and moderate physical activity^32^ for 72 and 24 h, respectively; refrain from alcohol for 24 h; and consume no food or beverage other than water for 8 h. Compliance with pretest restrictions was confirmed via questionnaire, and both sessions were performed in the morning between 0800 h and 1000 h in a room with an ambient temperature of ~21 °C. Participants wore standard clothing (briefs, shorts, and socks; total insulation = 0.12 clo^33^) for each session. At the PCP session, subjects’ height, mass, and waist circumference were measured using a calibrated stadiometer, scale, and Gulick tape measure, respectively. Body height and mass were used to calculate body mass index (mass (kg) divided by height squared (m)) and estimate body surface area with the DuBois and DuBois formula^34^.

Familiarization and PCP cold exposure methods were performed as described previously^27^, and similar equipment and subject setup details were implemented in the MRI session. In brief, two water-circulating blankets were secured around the participant and connected to a Blanketrol® III hyper-hypothermia system (Cincinnati Sub-Zero, Cincinnati, OH, USA). Blanket water temperatures were set to ~ 32 °C for an initial thermoneutral period, and temperatures were then decreased to achieve the target cooling profile for the session. Participants provided thermal sensation (e.g. “Neutral”, “Cold”, “Very Cold”) and shivering feedback in real-time with a keypad connected to a thermoesthesia Graphical User Interface (tGUI)^35^ throughout each session. Familiarization and PCP cooling profiles were designed to introduce the participant to the test environment and to identify the participant’s shiver threshold – the water temperature that elicited sustained shivering (>1 min duration as self-reported on the tGUI tool)^27^. For the MRI session, the cooling profile was individualized to maximize cold exposure and minimize shivering (Fig. 1). In general, blanket water temperatures were lowered from thermoneutral to 6 °C above the participant’s shiver threshold before cooling continued to 3 °C above shiver threshold for the remaining duration of the protocol (59.5 ± 6.5 min total cold exposure, depending on the schedule constraints of the imaging session). To personalize cooling progression, we asked the participant (via the MRI telecom system) in 8 min intervals (or phases) to self-report his ability to tolerate the temperature without shivering. If shivering occurred, water temperature was increased to 6 °C above shiver threshold for a phase or until shivering ceased. Set and actual water temperatures were logged every 30 s to a laptop computer (Blanketrol® III Data Export Software). Blanketrol® III and tGUI data were synchronized to image acquisition time stamps and summarized offline^36^.

### MRI data acquisition

MRI data were acquired using a Philips Achieva 3T scanner equipped with a 16-channel neurovascular coil (Philips Healthcare, Best, The Netherlands) (Fig. 1). Three plane localizers and high-resolution T_2_-weighted images were obtained of the neck and upper torso to assist in planning fat-water MRI scans of the supraclavicular region. The fat-water MRI sequence, summarized in Table 1, was performed at thermoneutral (acquisition 1) and between 20 to 28 times per participant during cold exposure. Image analyses were completed offline using custom scripts written in MATLAB (Mathworks, Natick, MA).

**Table 1:** Fat-Water Magnetic Resonance Imaging Sequence Parameters.

Imaging Parameter	Value
Pulse Sequence	3D multiple fast field echo
Coil	16-channel neurovascular receive coil
Orientation	Axial
Number of Slices	15
Axial In-Plane Field of View	530 × 200 mm
Acquired Voxel Size	1.25 × 1.25 × 4.00 mm
Acquisition Time	115.7 s
Repetition Time	17 ms
Number of Echoes	18 (3 × 6 interleaved sets)
First Echo Time	1.395 ms
Effective Echo Time	0.737 ms
Flip Angle	5°
Water-Fat Shift	0.505 pixels

No contrast agents were used. Data were acquired under normal breathing conditions. Preparation phases for each scan included first order linear B_0_ shimming and center frequency optimization.

### MRI data processing

#### Fat-water separation

**Figure 2:**
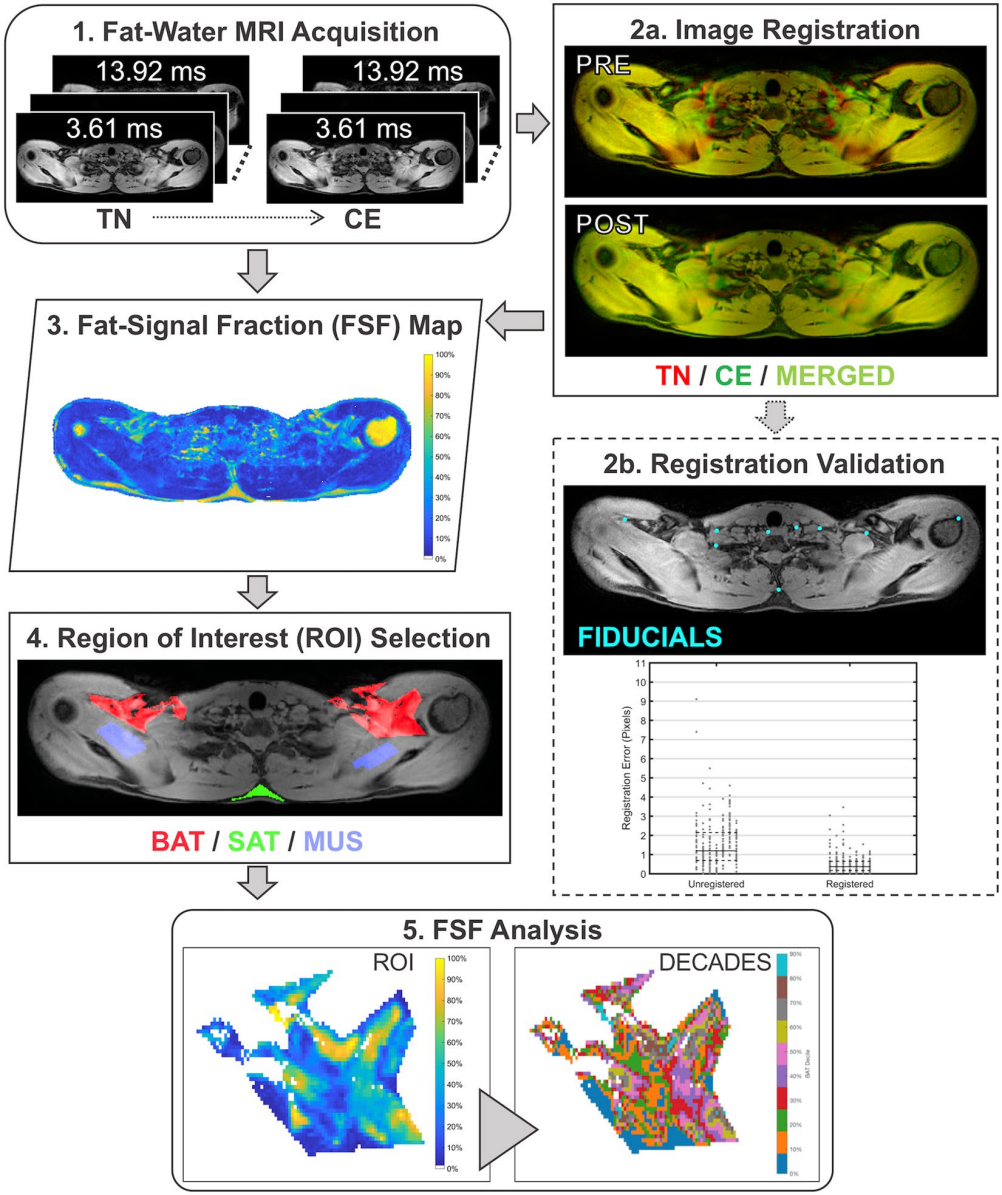
Overview of magnetic resonance imaging (MRI) processing methods. **Process 1**. Fat-water MR images were acquired during thermoneutral (TN) and cold exposure (CE). Exemplary magnitude images for echo times 3.61 ms, 4.34 ms, and 13.92 ms are shown. **Process 2a**. TN and CE images were co-registered with a three-dimensional nonrigid registration technique. **Process 2b**. A nine-point fiducial mapping approach was used to validate image registration. The in-plane Euclidean distance between fiducials was reduced following registration resulting in a median error of less than one pixel. **Process 3**. Registered fat-signal fraction (FSF, %) maps were derived from the fat-water MR images and corresponding three-dimensional deformation fields. **Process 4**. Regions of interest (ROIs) were manually delineated in brown adipose tissue (BAT, red), subcutaneous adipose tissue (SAT, green), and lean muscle (MUS, blue). **Process 5**. FSF analysis considered all voxels in each tissue ROI and included an FSF decade grouping approach. An example of a BAT FSF map (left) is shown with its corresponding FSF decade map (right).

Fat-water image reconstruction was completed using procedures similar to those described in our previously published method^19^ and are summarized briefly here. Initial processing steps removed the first echo of each six-echo train (Fig. 2, process 1, “Fat-Water MRI Acquisition”) to reduce potential phase contamination from the eddy currents in the complex fat-water signal^37^. Fat-water separation was then performed using a complex, three-dimensional optimization algorithm^38^ with a seven-peak spectral fat model that has been validated for 3T scanner field strength across a range of fat fractions^39^. We conducted simulation studies that demonstrated that it was not necessary to account for potential temperature-induced changes in the water proton resonance frequency within the brown adipose tissue when estimating the FSF (see Supplementary Materials). FSF parameter maps were calculated from the fat and water magnitude images while considering the dominant signal (i.e. water or fat) of each voxel^19^, and background voxels representing noise were removed.

#### Image registration

Image volumes were co-registered for each participant to compensate for shifts in body position during the MRI cooling protocol (Fig. 2, process 2a, “Image Registration”). Using the water magnitude images, the three-dimensional spatial correspondences between the first echo of the initial (acquisition 1) and subsequent (acquisitions 2–21…29) images were obtained (MATLAB function *imregdemons*). The resultant three-dimensional deformation fields were then applied (MATLAB function *imwarp*) to each FSF parameter map to complete registration (Fig. 2, process 3, “Fat-Signal Fraction (FSF) Map”). To reduce the impact of registration artifacts on FSF analyses, analysis of the image volumes was constrained to slices 6 to 13, an approximate anatomical region covering the distal portion of the neck through the apex of the lungs.

Image registration results were validated using a control point mapping technique (Fig. 2, process 2b, “Registration Validation”). Nine control points or fiducials were manually selected (B. M. D.) in four slices in the thermoneutral, unregistered cold exposure, and registered cold exposure images. The final cold exposure image was selected for each participant because it reflected the cumulative effect of all body motion throughout the study. The investigator was blinded to the image type (i.e. thermoneutral, unregistered, or registered), and control points were placed at reproducible anatomical landmarks and near possible brown adipose tissue depots. The in-plane Euclidean distance was calculated for the control points before and after registration, and registration errors were summarized across all subjects.

#### Region of interest (ROI) definition

Supraclavicular brown adipose tissue, subcutaneous adipose tissue, and muscle regions of interest (ROIs) were delineated manually (C. L. C.) and slice wise on the right and left sides of the body on the thermoneutral FSF map (Fig. 2, process 4, “ROI Selection”). Preliminary analysis did not suggest left-right differences; therefore, ROIs were combined to form a single bilateral ROI for each tissue type. The supraclavicular ROI was defined to include adipose tissue located between the clavicle and scapula with care taken to avoid bone marrow and areas adjacent to the lungs. When selecting the brown adipose tissue ROI boundary, the range of displayed FSF values was constrained between 30 and 80% to limit inclusion of muscle and subcutaneous adipose tissue in areas where the tissues were adjacent. All ROIs were eroded once to reduce the impact of partial volume artifacts (signal averaging of multiple, overlapping tissues in a single volume) on the calculated FSF value, and voxels containing erroneous fat-water separation results (FSF < 0 or > 100%) were masked. ROIs were then applied to the respective slice in the remaining co-registered images.

#### FSF analysis

FSF values were summarized over all slices in each ROI. Due to the lack of an established FSF range for brown adipose tissue^13^, we considered three threshold options for the ROI: 0–100%, 40–100%^40–42^, and 50–100%^19,43^. All FSF values (0–100%) were included in the subcutaneous adipose tissue and muscle ROIs. We also implemented an FSF decade grouping approach to further explore the apparent heterogeneity of lipid content within brown adipose tissue (Fig. 2, process 5, “FSF Analysis”). In the thermoneutral image (acquisition 1), voxels in the ROI of each tissue type were assigned to an FSF decade {0–10%}, {10–20%},… {90–100%}, where {10–20%} indicates FSF values greater than or equal to 10% and less than 20%. A voxel’s identity in the FSF decade was maintained for each subsequent image, and FSF decades with <60 voxels were excluded from summary analyses to ensure stable estimates of mean FSF. Bootstrap resampling of a population of >4000 voxel-wise FSF values was used to determine a minimum ROI size of 60 voxels was sufficient to estimate mean FSF within ±5%.

**Figure 3:**
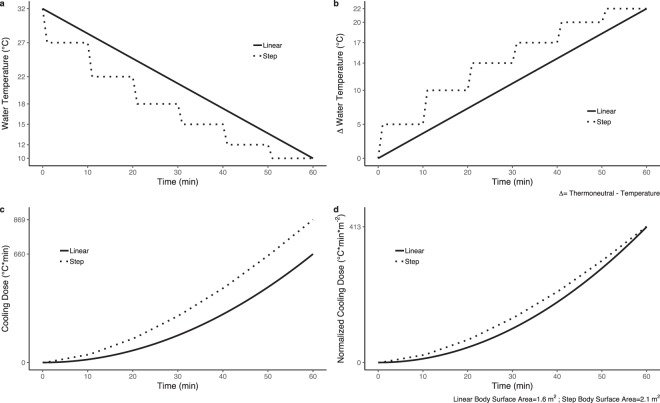
Conceptual model illustrating the calculation of normalized cooling dose, a construct we created to standardize reporting of personalized cooling protocols. Simulated water temperature (°C) vs. time (min) plots were created to compare two protocols of equal duration that begin (32 °C) and end (10 °C) at the same temperatures but follow linear (solid line) or step (dashed line) cooling gradient profiles (**a**). To calculate normalized cooling dose, the following steps are completed: (1) relative water temperatures (°C) are expressed as the change (Δ) in temperature from the starting or thermoneutral temperature (**b**), (2) cooling dose (°C*min) is calculated from the area under the relative water temperature curve (**c**), and (3) normalized cooling dose (°C*min*m^−2^) is found by dividing cooling dose by body surface area to account for differences in participant body size (**d**).

FSF values were averaged as a function of image acquisition number; as a function of normalized cooling dose – a construct we developed to account for temperature, temporal, and body size differences associated with individualized cooling (Fig. 3); and as a function of thermal sensation. Normalized cooling dose, Eq. (1), was calculated for each participant using the recorded cooling profile data:

1 $$\begin{array}{llll}Normalized\,Cooling\,Dose(t) & = & \frac{{\int }_{{t}_{1}}^{{t}_{2}}Relative\,Water\,Temperature(t)\,dt}{Body\,Surface\,Area} & [^\circ {\rm{C}}\,\ast \,{\rm{\min }}\,\ast \,{{\rm{m}}}^{-2}]\end{array}$$

where relative water temperature represented the difference in blanket water temperature from the thermoneutral temperature (T_0_ − T(*t*)), and t_2_ − t_1_ was the time interval of interest (e.g. the time between fat-water MRI acquisitions for the MRI session). Thermal sensation values were extracted from the MRI-synchronized tGUI data.

### Statistical analysis

Nonparametric tests were used for all analyses due to the small sample size (*n* = 7) of the study, and significance was defined as *P* < 0.05. Paired samples Wilcoxon signed rank tests were performed to compare PCP and MRI session conditions and to compare thermoneutral (acquisition 1) and cold exposure (acquisition 20) mean FSF for each ROI and FSF decade. Data for these comparisons are presented as the difference in mean FSF (thermoneutral – cold exposure) and the bootstrapped (1000 samples) nonparametric 95% confidence interval (CI) for the difference in the mean, unless otherwise noted. In addition, Spearman’s rank correlation coefficients (ρ, *n* = 179 observations) were calculated to test the relationships between mean FSF, normalized cooling dose, and thermal sensation for each brown adipose tissue FSF decade. Statistical analyses were performed in R Studio (version 1.0.153; R Studio, Boston, MA, USA).

## Results

### Subject characteristics and session conditions

Eight men completed the study at Vanderbilt University Medical Center in Nashville, TN between December 2016 and June 2017. Data for one volunteer were excluded from analyses due to excessive motion during the MRI session. The general physical characteristics of the volunteers and session environmental conditions were unremarkable (Table 2). Shiver threshold water temperature and normalized cooling dose varied, by design, across individuals during the PCP session. Although unintended, cooling dose (*P* = 0.94) and normalized cooling dose (*P* = 0.94) did not differ significantly between PCP and MRI sessions.

**Table 2:** Summary of Participant Characteristics and Session Conditions.

Characteristic	Value
*n*	7
Age (years)	26.7 ± 3.4 (22 to 33)
Height (cm)	170.6 ± 6.5 (162 to 180.3)
Mass (kg)	70.8 ± 10.2 (55.8 to 86.4)
Body Mass Index (kg/m^2^)	23.6 ± 2.5 (19.3 to 27.7)
Body Surface Area (m^2^)	1.82 ± 0.15 (1.64 to 2.03)
Waist Circumference (cm)	83.1 ± 5.2 (75.8 to 90.4)
***Perception Based Cooling (PCP) Session***	
Outdoor Temperature (°C)	8.5 ± 11.6 (−5 to 26)
Outdoor Humidity (%)	72.9 ± 15.9 (48 to 89)
Indoor Temperature (°C)	20.7 ± 1.9 (17 to 23)
Indoor Humidity (%)	41.7 ± 17.8 (21 to 70)
Shiver Threshold (°C)	15.6 ± 2.4 (13 to 20)
Cooling Dose (°C*min)	698.6 ± 213.5 (400.8 to 1078.3)
Normalized Cooling Dose (°C*min*m^−2^)	383.5 ± 117.0 (243.7 to 604.4)
***Magnetic Resonance Imaging (MRI) Session***	
Outdoor Temperature (°C)	10.2 ± 9.7 (−4 to 22)
Outdoor Humidity (%)	85.0 ± 13.0 (66 to 100)
Indoor Temperature (°C)	21.1 ± 0.76 (20.4 to 22)
Indoor Humidity (%)	48.8 ± 4.7 (41.8 to 51.8)
Cooling Dose (°C*min)	665.9 ± 130.1 (406.1 to 758.3)
Normalized Cooling Dose (°C*min*m^−2^)	366.2 ± 71.5 (246.9 to 436.3)

Values are means ± s.d. (Minimum to Maximum). Body surface area was calculated using the formula of DuBois and DuBois^34^. Environmental conditions were recorded at the start of the PCP and MRI cooling sessions. Indoor MRI conditions were not available for three sessions. Shiver threshold was the water temperature that elicited sustained shivering (>1 min in duration) in a participant during the PCP session^27^. Cooling dose was calculated as the area under the relative water temperature versus time curve recorded for each participant during the PCP and MRI sessions. Cooling dose was divided by body surface area to account for differences in participant body size.

### Image registration validation

The Euclidean distance between control point locations in the target and unregistered images had a median value of 1.19 pixels (interquartile range, IQR: 1.46 pixels; Fig. 2, process 2b, “Registration Validation”). Registration reduced the median Euclidean distance to 0.37 pixels (IQR: 0.46 pixels). Notably, the final registration error of less than one pixel supports the use of quantitative pixel-by-pixel comparisons of mean FSF changes.

### Effect of cold exposure on tissue lipid content

#### Region of interest

**Figure 4:**
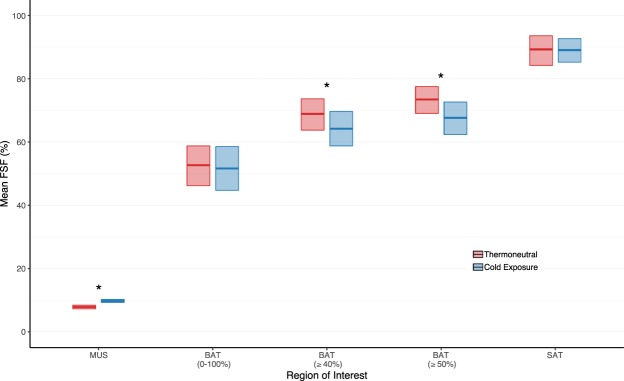
Applying a fat-signal fraction (FSF, %) threshold altered the interpreted effect of cold exposure on brown adipose tissue (BAT). Comparison of thermoneutral (red) and cold exposure (blue) mean FSF for muscle (MUS), BAT, and subcutaneous adipose tissue (SAT). Mean FSF for BAT were calculated using three threshold conditions: 0–100%, 40–100%, and 50–100%. The center line in each box indicates the mean, and the top and bottom of the box show the 95% bootstrapped confidence intervals for the mean (1000 samples). Wilcoxon signed rank tests were performed to compare thermoneutral and cold exposure conditions (*n* = 7): *P < 0.05.

Imposing an FSF threshold altered the interpreted effect of cold exposure on brown adipose tissue lipid content (Fig. 4). When considering the entire FSF range (0–100%), fat content in brown adipose tissue (−1.0%, 95% CI: −10.2 to 7.6%, *P* = 0.11) and subcutaneous adipose tissue (−0.2%, 95% CI: −6.2 to 5.9%, *P* = 0.38) did not significantly change while muscle tissue (1.9%, 95% CI: 1.1 to 2.7%, *P* = 0.016) exhibited an increase in fat content after exposure to cold. Setting the FSF threshold to 40–100% (−4.7%, 95% CI: −12.0 to 2.5%, *P* = 0.016) and 50–100% (−5.8%, 95% CI: −12.7 to 0.8%, *P* = 0.016) for the ROI, however, revealed a significant reduction in lipid content in brown adipose tissue in response to cold exposure. As expected, mean FSF differed (*P* = 0.016) between brown adipose tissue (0–100% FSF; mean ± standard deviation (s.d.): 52.7 ± 9.5%) and subcutaneous adipose tissue (mean ± s.d.: 89.3 ± 6.7%) at thermoneutrality.

#### FSF decades

**Figure 5:**
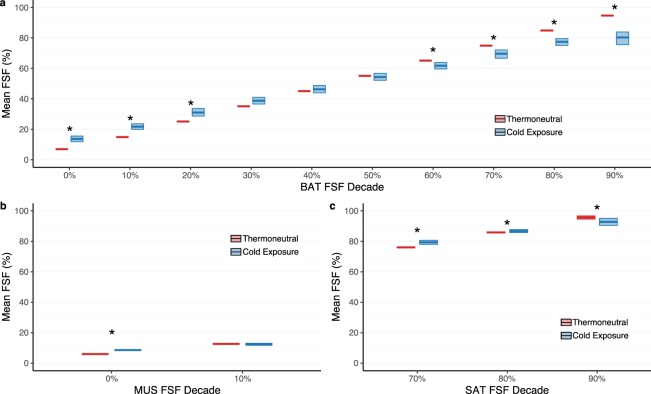
Brown adipose tissue (BAT) lipid content underwent differential changes in mean fat-signal fraction (FSF, %) in response to cold exposure with significant increases at lower FSF decades (0–20%) and significant reductions in lipid content at the higher FSF decades (60–90%). Comparison of thermoneutral (red) and cold exposure (blue) mean FSF for FSF decades in BAT (**a**), muscle (MUS; **b**), and subcutaneous adipose tissue (SAT; **c**). Image voxels included in each tissue region of interest were assigned to an FSF decade according to the voxel’s initial FSF value (i.e. thermoneutral). For example, an initial FSF value of 33% was assigned to the 30% FSF decade. A minimum of 60 voxels were required for the FSF decade to be included in the summary analysis. The center line in each box indicates the mean, and the top and bottom of the box show the 95% bootstrapped confidence intervals of the mean (1000 samples). Wilcoxon signed rank tests were performed to compare thermoneutral and cold exposure conditions (*n* = 7): *P < 0.05.

Brown adipose tissue exhibited a varied pattern of lipid loss and uptake in response to cold exposure (Fig. 5a). Mean FSF decreased significantly in FSF decades with high initial lipid contents (60%, *P* = 0.047; 70–90%, *P* = 0.016) with the largest decrease noted in the 90% decade (−14.4%, 95% CI: −10.5 to −19.2%). In contrast, FSF decades with low initial amounts of lipid (0–20%; *P* = 0.016) demonstrated a significant increase in mean FSF predominantly in the 0% decade (6.7%, 95% CI: 4.8 to 8.6%). Brown adipose tissue FSF decades in the middle lipid range (30%, *P* = 0.078; 40%, *P* = 0.47; 50%, *P* = 0.69) were not significantly altered following cold exposure. In skeletal muscle (2.7%, 95% CI: 2.1 to 3.1%, *P* = 0.016; Fig. 5b) and in subcutaneous adipose tissue (−2.9%, 95% CI: −0.4 to −5.6%, *P* = 0.016; Fig. 5c) small but significant increases and decreases in FSF were detected in the extreme FSF decades (i.e. 0% and 90%), respectively. Interestingly, cold exposure did not change mean FSF in the 10% decade in muscle (*P* = 0.69) and unlike brown adipose tissue, mean FSF increased in the 70% (3.4%, 95% CI: 2.0 to 4.7%, *P* = 0.031) and 80% (0.87%, 95% CI: −0.18 to 2.0%, *P* = 0.031) decades of subcutaneous adipose tissue.

#### Relationship with normalized cooling dose and thermal sensation

**Figure 6:**
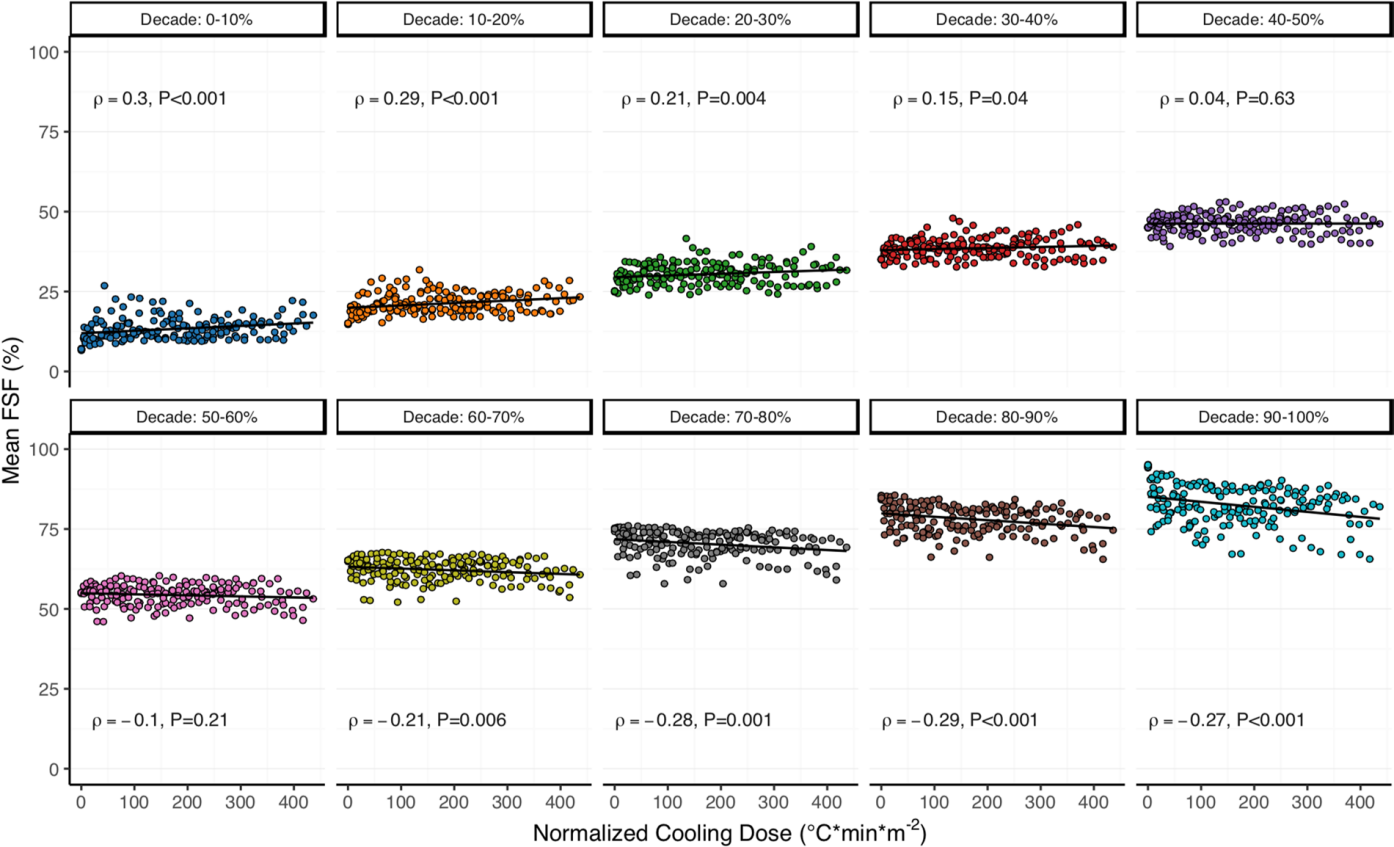
Cold-induced changes in mean fat-signal fraction (FSF, %) in each brown adipose tissue (BAT) FSF decade (0–10%: blue; 10–20%: orange; 20–30%: dark-green; 30–40%: red; 40–50%: purple; 50–60%: pink; 60–70%: lime-green; 70–80%: gray; 80–90%: brown; 90–100%: teal) correlated with normalized cooling dose (°C*min*m^−2^). Data points indicate the individual mean FSF and normalized cooling dose calculated for each fat-water magnetic resonance image (i.e. 21–29 data points for each of the 7 participants). Least squares best-fit lines are overlaid on the mean FSF data. Correlation (ρ) between mean FSF and normalized cooling dose was evaluated with the Spearman’s rank test (*n* = 179).

**Figure 7:**
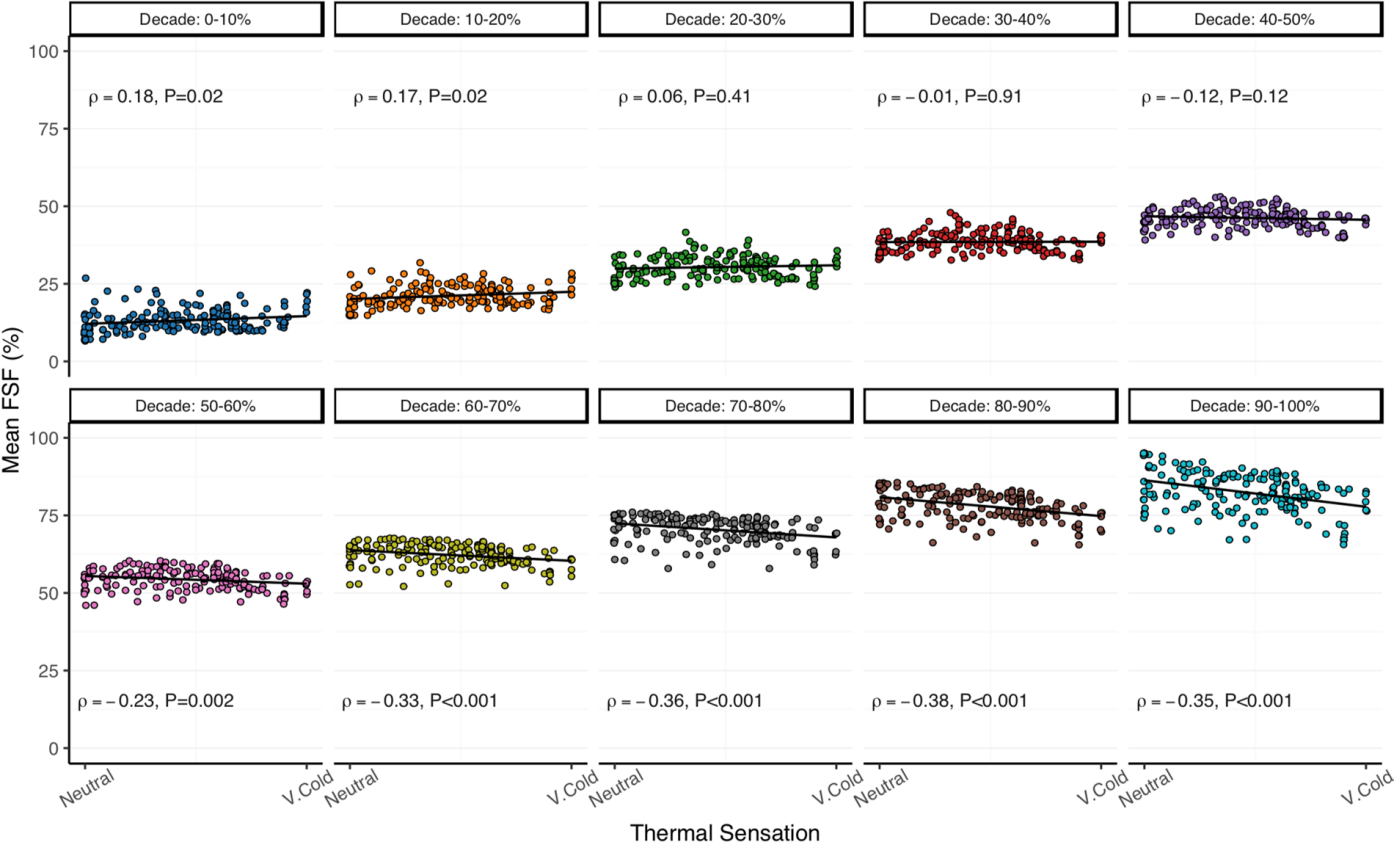
Cold-induced changes in mean fat-signal fraction (FSF, %) in each brown adipose tissue (BAT) FSF decade (0–10%: blue; 10–20%: orange; 20–30%: dark-green; 30–40%: red; 40–50%: purple; 50–60%: pink; 60–70%: lime-green; 70–80%: gray; 80–90%: brown; 90–100%: teal) correlated with thermal sensation (arbitrary units). Thermal sensation was recorded on a continuous integer scale (Neutral = 50 and Very Cold (V. Cold) = 0) via a thermoesthesia graphical user interface^35^. Data points indicate the individual mean FSF and thermal sensation value acquired for each fat-water magnetic resonance image (i.e. 21–29 data points for each of the 7 participants). Least squares best-fit lines are overlaid on the mean FSF data. Correlation (ρ) between mean FSF and thermal sensation was evaluated with the Spearman’s rank test (*n* = 179).

Changes in brown adipose tissue lipid content correlated with normalized cooling dose (Fig. 6) and thermal sensation (Fig. 7). Mean FSF in high (60, 70, 80, and 90%) and low (0, 10, and 20%) FSF decades demonstrated opposing weak negative and positive relationships with normalized cooling dose, respectively. Similar relationships existed between mean FSF and thermal sensation; however, the relationship was stronger in the high (50, 60, 70, 80, and 90%) and weaker in the low (0 and 10%) FSF decades. Mean FSF was not correlated with either normalized cooling dose (30, 40, and 50%) or thermal sensation (20, 30, and 40%) for FSF decades in the middle lipid range.

## Discussion

The present study highlights the dynamic response of human brown adipose tissue to cold exposure and the unique ability of fat-water MRI to quantify these changes as functions of time. Our data revealed a diverse distribution of high and low lipid areas interspersed throughout the supraclavicular brown adipose tissue depot. Upon cold exposure, areas initially rich with lipid exhibited an immediate and dramatic reduction in FSF while a simultaneous, opposing increase in FSF occurred in areas with low initial amounts of lipid. Lipid loss and uptake were also detected in subcutaneous adipose tissue and skeletal muscle near the neck, respectively. Together, these findings provide further support to the suggestion of a coordinated response between brown adipose tissue, subcutaneous adipose tissue, and skeletal muscle to a variety of stressors including exercise and cold exposure^11,44–47^.

Defining brown adipose tissue with different FSF thresholds influenced ROI-based analysis of supraclavicular brown adipose tissue lipid content. When including all voxels, brown adipose tissue FSF was within the range of previously reported values, which have been as low as 23% in infants^48^ and as high as 94% in elderly adults^49^. Brown adipose tissue lipid content was also significantly lower than subcutaneous adipose tissue during thermoneutral conditions, a finding consistent with other fat-water MRI studies^19,43,49–51^. Setting different brown adipose tissue FSF thresholds, however, altered both the mean FSF value and the significance of the effect of cold exposure on brown adipose tissue lipid content – conflicting variabilities noted in other FSF analyses^13^. Lower and upper FSF thresholds are thought to remove voxels containing higher ratios of low (e.g. muscle, connective tissue, or blood vessels) and high (e.g. white adipocytes) fat tissues, respectively, to reduce the impact of partial volume artifacts on MRI-based detection of brown adipose tissue^22,52^. An optimal FSF threshold, however, remains unclear^13,26^ and is likely to vary with the age and body composition of the study population^48,53–55^. Excluding FSF values could also obfuscate functional characteristics of mixed beige, brown, and white adipocytes located in supraclavicular adipose in adult humans^56–58^. For these reasons, we created a voxel-wise, FSF decade analysis which allowed us to compare the heterogeneous morphology of brown adipose tissue to subcutaneous adipose tissue and skeletal muscle.

The significant cold-induced net loss in lipid content in brown adipose tissue FSF decades with high initial FSF parallels intracellular processes associated with thermogenesis. Although overlap is common, FSF values > 80% have been associated with a white rather than a brown adipose tissue phenotype^13,50,52^, resulting in their exclusion from some analyses^23,59^. We found it notable, therefore, that high FSF decades (60% to 90%) in the supraclavicular depot demonstrated a profound loss in fat content several fold greater than has been previously reported^19,23,40,42,43,60^. Moreover, these trends were not mirrored in subcutaneous adipose tissue, which showed a small decrease in FSF in only the 90% decade. Additional biochemical and histological studies are necessary to discern definitively if the selected depot contained brown adipose tissue as well as if each FSF decade had a higher ratio of beige, brown, or white adipocytes. We can also not exclude partial volume effects of small vessels, nerves, or other non-adipose tissues^22,52^. We speculate, however, that the measured shifts in lipid content indicate lipolysis of intracellular triglycerides to fuel brown adipose tissue thermogenesis^6–10^. Lipid loss in both the brown^61^ and subcutaneous adipose tissue^9^ could also reflect a cold-induced release of fatty acids into the circulation for use elsewhere in the body^44,62^.

Further, increases in net lipid content in the low FSF voxels of the brown adipose tissue suggests a replenishing of intracellular lipid pools to maintain thermogenesis. Cold exposure has been shown to induce a simultaneous uptake of fatty acids, glucose, and other substrates from the circulation into brown adipose tissue to generate lipid droplets for further use^46,63–65^. Our data suggest uptake of fatty acids and/or triglyceride synthesis in brown adipose tissue with low fat contents at thermoneutrality. These findings are in line with recent PET-CT data by Din *et al*.^17^, in which brown adipose tissue regions with higher radiodensity (i.e. lower lipid contents) had a greater uptake of non-esterified fatty acids. Corresponding increases in FSF in skeletal muscle and subcutaneous adipose tissue also support a cold-stimulated uptake of fatty acids^7^ for combustion or storage, respectively^45^.

It remains unclear if lipids in the middle FSF decades (30–60%) in brown adipose tissue underwent a change in response to cold. The similarity of this FSF range to that found in brown adipose tissue in human infants^48,56^ implies that the region has a high thermogenic capacity and subsequent demand for intracellular free fatty acids in response to cold exposure^2,63^. Stable lipid content measurements, however, may indicate a dynamic steady-state between the intracellular rates of lipolysis and fatty acid re-esterification^62^. Noninvasive imaging methods are needed to quantify the direct amount of heat produced by brown adipose tissue^60^ to resolve if adipose tissue with FSF values in this middle range contribute to cold-induced thermogenesis.

Lipid mobilization within the brown adipose tissue depot occurred rapidly in response to a cold stimulus. With progressive cold stress, thermoreceptors in the skin initiate a feedforward response via the hypothalamus to activate thermoregulatory processes including peripheral vasoconstriction and brown adipose tissue thermogenesis to maintain core body temperature^66^. Our findings support this, as both normalized cooling dose and thermal sensation, a surrogate measure of peripheral vasoconstriction^27^, correlated with the dynamic changes in brown adipose tissue lipid content. However, the observation that normalized cooling dose and thermal sensation explained at most 15% of the variance in FSF suggests that while personalized cooling limited the incidence of shivering, other individual responses to cold exposure^67^ or aspects of the imaging protocol continue to contribute to the variability in brown adipose tissue FSF. Future work should consider acquiring images during a prolonged thermoneutral period to establish the baseline repeatability of tissue FSF values. Extending the duration or severity of the cooling procedure also remains an area of interest to determine if the changes in lipid content in the low- and high-FSF decades reach a steady-state or if these alterations continue while brown adipose tissue is maximally activated.

The discussion above presumes that the observed changes in FSF are due primarily to changes in lipid content. One notable competing explanation is an increase in perfusion. Brown adipose tissue is highly vascularized, allowing for the dissipation of heat and the transport of free fatty acids to the cells^11,68^; and previous studies have reported a two-fold increase in perfusion to brown adipose tissue in response to cold^69^. It is conceivable that vasodilation and a resulting displacement of the tissue around vessels would increase the water signal fraction of the voxels. For several reasons, we argue that this process could not be a quantitatively important contribution to the FSF changes that we observed. First, Blondin *et al*.^6^ found that intracellular triglycerides were the primary substrate for brown adipose tissue, with no changes in perfusion. Also, Lundstrom *et al*.^40^ found that changes in FSF due to cold exposure persisted following reheating, which is inconsistent with the typically rapid on- and off-kinetics of perfusion. Lastly, an effect based principally on vasodilation is inconsistent with the data presented in Figs 5–7. In thermoneutrality, the mean FSF in the 90–100% decile was 95%; at the end of cold exposure, the mean FSF in this decade was 81.0%. Presuming that half of the water signal originated in blood at thermoneutrality (a blood volume fraction of 2.5%), then the blood volume fraction would need to increase to 16.5% of total tissue volume to explain the reduction of mean FSF to 81%. An effect based principally on vasodilation also could not explain the absence of net change in FSF in the middle decades, the increase in FSF in the lower decades, the varied responses within subcutaneous adipose tissue and muscle, or the monotonically changing FSF values over a period of 45–60 minutes. For these reasons, we conclude that the observed changes in FSF are due entirely or almost entirely to changes in lipid content.

A strength of this study is the use of personalized cooling during image acquisition, which allows for changes in brown adipose tissue lipid content to be reported with respect to the amount of cold exposure. This approach shows promise for investigating fundamental questions about brown adipose tissue physiology including: the extent of activation at a given cold stress and whether the magnitude of cold exposure associated with maximal activation differs between subjects^70^. A key challenge for these future studies, however, is the need for a standardized definition of cold exposure in the context of individualized protocols that vary – by design – in duration, temperature, and cooling gradient to minimize shivering for each participant. Ideally, precise measures of inlet and outlet water temperature would enable cold stress to be expressed as the change in temperature between the body and the ambient environment, but these types of data may not be possible without custom equipment^6,23^. Here, we introduce the concept of cooling dose as a starting point to address the need for a standardized definition of cold exposure. Cooling dose is simple to calculate using time and temperature data reported from many commercial cooling systems. Incorporating anthropometric and physiological factors (e.g. body surface area, subcutaneous fat thickness, or resting metabolic rate) can further standardize cooling dose to better describe the complex nature of human thermal regulation^67^. We normalized cooling dose using body surface area because we believed it provided a better geometrical representation of the human body than body mass index, and it could easily be estimated with body height and mass, descriptive characteristics measured in most human studies. Testing a larger population with greater diversity than tested here is necessary to refine the normalized cooling dose unit, and in general, to determine if the observed changes in brown adipose tissue lipid content differ with sex, age, body composition, or disease state.

Direct comparisons between our findings and previous fat-water MRI studies are difficult due to diverse experimental procedures, imaging sequences, and data analysis techniques. Extending recent standardization efforts for PET-CT imaging of brown adipose tissue to include MRI-based methods would improve inter-study comparability and establish best practices as techniques are validated and optimized^70^. For example, using our imaging sequence we can simultaneously calculate co-registered brown adipose tissue FSF and T_2_* maps. T_2_* is sensitive to iron content and has been proposed as a metric to differentiate brown and subcutaneous adipose tissue^13^. However, we did not include T_2_* data in the present analysis because recent findings from Franz *et al*.^71^ indicated a 20-echo sequence is required for accurate analysis of T_2_* in adipose tissue. Additional limitations of the present study also offer potential for improvement. Acquiring images during breath-holds, a possibility with faster fat-water MRI sequences, could reduce image registration errors that cannot be excluded in this data obtained under free breathing conditions. Further, incorporating automated methods to segment adipose tissue^41^, opposed to the manual delineation of ROIs, could improve the reliability and validity of MRI-based studies of brown adipose tissue. If automated segmentation is not feasible, setting a lower FSF threshold equal to the mean FSF in muscle could also reduce inclusion of skeletal muscle voxels in adipose tissue ROIs.

In conclusion, we found that supraclavicular brown adipose tissue in healthy, adult men contained a heterogeneous mixture of high- and low-lipid areas. Immediately in response to a cold stimulus, these zones underwent differential changes in total lipid content that mirrored sympathetically-mediated, intracellular processes of lipolysis and uptake of fatty acids associated with brown adipose tissue thermogenesis. Corresponding shifts in lipid content in subcutaneous adipose tissue and skeletal muscle highlight the potential of fat-water MRI to investigate the transport and metabolism of lipid within and possibly between brown adipose tissue and other tissues and organs. Finally, our findings support standardized brown adipose tissue FSF thresholds for future analyses to better elucidate how diverse changes in human brown adipose tissue morphology and physiology relate to metabolic health and disease.

## Supplementary information

Supplementary Material

## Data Availability

Summarized data and code to reproduce the figures and statistical analyses reported in this article are available for public download at http://github.com/ccoolbaugh/SciRep-Coolbaugh-2019 ^72^. Raw MRI data generated during the current study are not available as the data have not explicitly been authorized for public release by the Vanderbilt University Medical Center Institutional Review Board. Code is also accessible to run the tGUI tool^35^ and to assist with summarizing individualized cooling protocol data^36^.
