# Supplementary material for: Cold exposure induces dynamic, heterogeneous alterations in human brown adipose tissue lipid content
Source: Sci Rep. 2019 Sep 19;9:13600. doi: 10.1038/s41598-019-49936-x (PMC6753098; doi:10.1038/s41598-019-49936-x)
Supplement: Supplementary file 1 — Supplementary Material [file 41598_2019_49936_MOESM1_ESM.docx]

***Supplementary Material***

**Cold exposure induces dynamic, heterogeneous alterations in human brown adipose tissue lipid content**

Crystal L. Coolbaugh, Bruce M. Damon, Emily C. Bush, E. Brian Welch, and Theodore F. Towse

**Purpose**

The water proton resonance frequency is inversely related to temperature, but fat/water separation algorithms ignore this shift. It is therefore conceivable that the failure to account for this shift when estimating fat-signal fraction (FSF) values could introduce error into these estimates. The goal of these simulations was the estimate this potential error across relevant ranges of FSF and temperature.

**Methods**

**General Strategy** Simulation studies were conducted in Matlab R2018b, Update 2. A total complex signal at different echo times (**S_T_**), composed of known fat and water signals, was synthesized for varying FSF and temperature values. FSF values of [0.05, 0.15, 0.25…0.95] were assumed. For each FSF value, signals were synthesized for temperatures of [36.0, 36.2, 36.4…40.0] ⁰C. These temperature-dependent signals were then used to estimate the FSF, but under the assumption of a constant chemical shift for water. Finally, the differences between the estimated and FSF values were calculated.

**Formation of Signals** To synthesize the lipid protons’ signals, we first assumed the seven-peak model for the lipid protons’ chemical shifts (δ_L_) and relative peak areas A_L_ presented in Table S1.

| **Table S1.** ***Seven-peak model for lipids***. Chemical shifts are expressed in parts per million (ppm) and are relative to tetramethylsilane (= 0.0 ppm). | |
| --- | --- |
| **Chemical Shifts, δ_L_ (ppm)** | **Relative Peak Areas, A_L_** |
| 1.10 | 0.085 |
| 1.50 | 0.625 |
| 1.79 | 0.071 |
| 2.23 | 0.095 |
| 2.45 | 0.066 |
| 2.97 | 0.016 |
| 5.51 | 0.042 |

We then synthesized the lipid protons’ signal vector, **S_L_**, with the lipid signal at each *TE*

$\mathbf{s}_{\mathbf{L}}\left( \mathbf{TE} \right)= \mathrm{FSF} \sum_{L} e^{-i 2\pi TE \gamma B_{0} \left( \left( \delta_{L}-\delta_{W} \right)\cdot{10}^{-6} \right)} A_{L}$ [S1]

Where TE is the array of echo times used in the experimental studies, γ is the gyromagnetic ratio in Hz/Tesla, B_0_ is the field strength in Tesla (=3.0), and δ_W_ is the chemical shift of water (4.80 ppm at 37⁰C). In Eq. S1, δ_W_ is subtracted from δ_L_ because the center frequency of the MR spectrometer is placed on resonance for water during data acquisition. Multiplication by 10^-6^ is necessary because chemical shifts are specified in ppm.

The water signal vector, $\mathbf{S}_{\mathbf{W}}$, is defined at each *TE* as

$\mathbf{s}_{\mathbf{W}}\left( \mathbf{TE} \right)= {\left( 1-FSF \right) e}^{-i2\pi TE\gamma B_{0}\Delta T\left( -0.01\frac{ppm}{^{\circ}C} \right)\cdot{10}^{-6}}$ [S2]

Where ΔT is the assumed temperature change and the constant –0.01 ppm/⁰C describes the effect of temperature on the water proton resonance frequency. **S_T_** was calculated as the complex sum of **S_L_** and **S_W_**.

**FSF Estimation** A model matrix, **A,** was formed naïve to the effect of temperature. **A** contained unit signal representations of water and fat (**S_W_** and **S_L_**, respectively) but without considering the effect of temperature on δ_W_. The estimated FSF was then calculated as:

$\mathbf{B}= \left( \mathbf{A}^{H}\mathbf{A} \right)^{-1}\mathbf{A}^{H}\mathbf{S}_{\mathbf{T}}$ [S3a]

**B** contains the estimated weights of the fat and water signal components:

$\mathbf{B=}\left[ \begin{matrix} {\hat{\boldsymbol{W}}}_{\boldsymbol{W}} \\ {\hat{\boldsymbol{W}}}_{\boldsymbol{L}} \end{matrix} \right]$ [S3b]

The FSF was estimated as:

$\hat{\mathbf{FSF}}\mathbf{=}\frac{\left| {\hat{\boldsymbol{W}}}_{\boldsymbol{L}} \right|}{\left| {\hat{\boldsymbol{W}}}_{\boldsymbol{L}} \right|\boldsymbol{+}\left| {\hat{\boldsymbol{W}}}_{\boldsymbol{W}} \right|}$ [S4]

and its error was calculated as $\left( \hat{\mathbf{FSF}}\boldsymbol{-}\mathbf{FSF} \right)$.

**Results and Discussion**

Figure S1 shows the estimated FSF values for each known FSF value, across temperatures ranging from 36-40⁰C. For all FSF values, unaccounted elevations in tissue temperature cause FSF to be overestimated, while unaccounted decreases in tissue temperature cause FSF to be underestimated. The greatest effect, observed with FSF = 5% at a temperature of 40⁰C, was 0.49 percentage units (i.e. the biased FSF was 5.49%).

**Figure S1*. Simulated effect of unaccounted temperature changes on the estimation of FSF, across ranges of physiologically relevant temperature and FSF values.***

For low FSF values, these predicted effects of brown adipose tissue’s thermogenesis on the estimation of its FSF values are qualitatively consistent with the experimental observation of an increase in FSF during cooling. However, there is substantial quantitative disagreement in the predicted and experimental data: the predicted effects are unable to explain the 6.7 percentage unit increase in FSF that occurred in the lowest FSF decade. Also, these effects are both qualitatively and quantitatively inconsistent with the 14.4 percentage unit decrease in FSF that occurred in the highest FSF decade.

**Conclusion**

These simulations suggest that errors in FSF estimation caused by the failure of the fat/water separation algorithm to account for temperature-induced shifts in the water proton resonance frequency cannot explain the experimentally observed FSF changes.
